# Forms of Face-to-Face Victimization as Significant Correlates of General Online Victimization and Sexual Online Victimization

**DOI:** 10.3390/bs14060441

**Published:** 2024-05-24

**Authors:** Annis Lai Chu Fung, Yuxuan Zhang

**Affiliations:** Department of Social and Behavioural Sciences, City University of Hong Kong, Tat Chee Avenue, Kowloon, Hong Kong SAR, China; yuxuan.z@my.cityu.edu.hk

**Keywords:** face-to-face victimization, online victimization, sexual online victimization, adolescents

## Abstract

Research has shown that face-to-face victimization is a risk factor for the online victimization of adolescents, but no prior study has examined and compared four forms of face-to-face victimization (physical victimization, verbal victimization, social manipulation, and attacks on property) as significant correlates of general online victimization and sexual online victimization among adolescents. This original study involved 794 adolescents (483 males and 311 females), aged 12 to 18 years (M = 14.49, SD = 1.90) from four middle schools in Hong Kong. The participants completed a self-report questionnaire consisting of three parts: the Multidimensional Peer Victimization Scale, the Online Victimization Scale, and demographic items. Verbal victimization and social manipulation were found to be significant correlates of general online victimization; in contrast, physical victimization and attacks on property were significant correlates of sexual online victimization. These findings may help professionals and educators to develop effective prevention and intervention strategies for preventing the cycle of victimization between physical and online platforms as well as reducing the suicide risk and crises among at-risk victimized adolescents.

## 1. Introduction

The rapid evolution of network technology has led to an increased prevalence of youths engaging in various forms of online socialization and interaction [1], significantly heightening their possibility of online victimization [2,3]. Online victimization generally refers to the deliberate and repeated victimization of individuals through online electronic devices [4,5]. Among the various manifestations of online victimization, general online victimization stands out as a widely examined dimension, encompassing the broad categories of hurt and harassment received online without specific directionality [6]. In some studies, general online victimization was also categorized as nonsexual victimization, including online harassment, pressure to obtain personal information, happy slapping, etc. [7]. A survey conducted in the United States middle schools showed that 18% of the students reported experiencing cyberbullying [8]. Another national survey indicated that 6% of adolescents encountered online victimization in the preceding year [9]. Additionally, many researchers and practitioners have directed attention to sexual online victimization among teenagers, recognizing them as a particularly vulnerable demographic to suffer from it. Sexual online victimization involves unwanted sexual requests or information exposure in an online environment, such as unwanted exposure to sexual material, requests to engage in sexual activities, sexual solicitation, online grooming, sexual pressure, etc. [10] Approximately 39% of adolescents are susceptible to sexual victimization in cyberspace [11]. Empirical studies have demonstrated that 60% of teenage participants in Italy reported experiencing sexual online victimization [11]. In Spain, around 12.6% and 7.9% of teens reported encountering sexual solicitations and engaging in sexual interactions, respectively [1].

Face-to-face victimization, also known as offline victimization, encompasses the direct or indirect harm inflicted by others [5,12], including physical victimization, verbal victimization, social manipulation, and attacks on property [13]. In recent decades, the prevalence of face-to-face victimization among adolescents has been high. In 2018, The Organization for Economic Cooperation and Development (OECD) conducted a Program for International Student Assessment (PISA) across 71 countries, showing that 23% of school-age students experienced face-to-face victimization several times per month [14]. A survey in the United States covering more than 7000 students in grades 6–10 showed that among these teenagers, the rate of physical victimization was 12.8%, the rate of verbal victimization was 36.5%, and the relational victimization rate was 41% [15].

Notably, face-to-face victimization appears to constitute a significant risk factor for youth’s online victimization [16]. First, many previous surveys support this view. A study in 2007 revealed that nearly 73% of young individuals who reported experiencing online victimization had also encountered face-to-face victimization [17]. In a subsequent study in 2011, the co-occurrence of online victimization and face-to-face victimization increased to 96% among the sampled adolescents [9]. Second, the self-determination theory and the social compensation hypothesis can be integrated to offer a robust theoretical foundation for the close connection and predictive relationship between face-to-face victimization and online victimization among adolescents. From the perspective of the individual, the self-determination theory emphasizes people’s basic needs for autonomy, competence, and relatedness [18]. When these needs are not met, individuals are more likely to experience negative outcomes (e.g., emotional disorders) and become less prone to finding solutions. From the perspective of social interactions, the social compensation hypothesis demonstrates that those with weak social skills or who face real-life social barriers may turn to the internet to find alternative social channels to make up for their deficiencies in face-to-face social interactions [19]. These individuals try to establish and maintain social connections and receive emotional support from the cyber context, thus enhancing their relatedness with others. Therefore, face-to-face victims exhibiting psychological needs deficits and lacking social support may show greater engagement in online environments, increasing their risk of online victimization. Meanwhile, their interactions online may also reflect their real-life vulnerabilities, making them easier targets for cyberbullying [20]. Overall, past empirical research and corresponding theoretical foundations jointly support the hypothesis that face-to-face victimization is positively related to and predicts online victimization.

Certain demographic factors, such as age and gender, exhibit strong associations with online victimization. Regarding age, research indicates that children aged 2–9 years old report minimal instances of online victimization, while a positive correlation exists between the age of teenagers (10–17 years old) and their experience of online victimization [9]. Additionally, older youth face a higher risk of becoming involved in sexual online victimization [11]. Concerning gender, a general association with sexual online victimization has been observed, although views on this matter remain controversial. Some studies insisted that female adolescents face a higher risk of sexual online victimization, attributing it to their tendency to receive more information or messages related to sexual content [3,21]. However, other studies argued that male adolescents are more online victimized than females [11,22].

Until now, no prior studies examined the roles of sex, age, and four forms of face-to-face peer victimization (physical and verbal victimization, social manipulation, and attacks on property) in associating general online victimization and sexual online victimization among adolescents. The most similar study examined whether age, gender, and online risk behavior were significant correlates of sexual online victimization among Italian adolescents aged between 12 and 14 [11] but did not investigate different forms of face-to-face victimization as correlates, or compare correlates of general online and sexual online victimization. Similarly, two other related studies investigated the correlates of unwanted online sexual solicitation among Swedish pupils in Grades 7 to 9 [23] and among Taiwanese youth in Grade 10 [24] but did not compare significant correlates of general online and sexual online victimization. Other studies have explored the correlates of online harassment [22] and four types of cybercrime victimization/experiences (online harassment, hacking, identity theft, and receiving nude photos or explicit content) [25], but did not explore the roles of sex, age, or peer victimization in associating general online victimization and sexual online victimization among adolescents.

A meta-analysis by Pratt et al. [26] revealed that self-control is a significant correlation for online victimization, but the analysis did not include any studies comparing general online and sexual online victimization. Likewise, some studies have focused on investigating the correlates of sexual online victimization and found that an increased engagement with social networks could expose adolescents to behaviors that are highly related to online sexual victimization [3], and that sexting and solicitations are strong correlates of sexual online victimization [27], but they did not compare significant correlates of general online and sexual online victimization.

To the best of the authors’ knowledge, this is the first study aiming to fill the research gap mentioned above by comparing the significant correlates of general and sexual online victimization by investigating their relationships with face-to-face peer victimization (verbal and physical victimization, social manipulation, and attacks on property), as well as age and sex. It was hypothesized that different forms of face-to-face peer victimization were associated with a higher risk of general and sexual online victimization in adolescents and that the associations were moderated by the age and sex of the victim. The present study can enhance the understanding of the relationship between the four specific forms of victimization under the face-to-face platform, which is directly associated with online victimization in that it helps provide further signs and indicators for parents, educators, counselors, social workers, and psychologists to pay attention to among adolescents.

## 2. Materials and Methods

### 2.1. Participants

The participants in this study were 794 students (483 males and 311 females) aged 12 to 18 years (M = 14.49, SD = 1.90) from four middle schools in Hong Kong.

### 2.2. Procedures

Ethical approval was obtained from the Research Committee of the City University of Hong Kong. The authors randomly sent invitation letters to one middle school from each of the five geographical constituencies in Hong Kong (Hong Kong Island, Kowloon East, Kowloon West, New Territories East, and New Territories West) to ask for the school leaders’ approval to invite students to participate in an anonymous questionnaire survey about online victimization among adolescents in Hong Kong. The schools were selected from a list of 20 partner schools that took part in the author’s previous research project on school violence. All five schools accepted this invitation, but one school withdrew from the study before data collection. Ultimately, four schools joined the study with permission obtained from the schools’ administration.

All Middle 1 to 7 (Grades 7 to 13) students were invited to complete a questionnaire. They were clearly informed that the survey was anonymous. Parental consent and students’ assent were provided before the study commenced. All participants and their parents were assured that the data collected would remain confidential and be used for research purposes only. Ultimately, around 80% of parents and their children agreed and completed the survey.

The questionnaire was administered in an electronic format through Qualtrics, accessed by desktop computers, to groups of about 20 students each, in a classroom setting during regular school hours. The participants were requested not to discuss the content of the survey with other students during or after filling in the questionnaire.

### 2.3. Measures

The questionnaire consisted of three parts: the Multidimensional Peer Victimization Scale (MPVS) [13], the Online Victimization Scale (OVS) [6], and demographic items.

### 2.4. MPVS

The MPVS is a self-report scale to evaluate peer victimization experiences in adolescents. It consisted of 16 items, with four subscales: physical victimization, verbal victimization, social manipulation, and attacks on property. This study utilized the Chinese version of the MPVS [28]. Participants rated each item on a 3-point Likert scale to indicate the frequency of victimization they experienced during the previous school term (0 = never, 1 = once, 2 = twice or more). The Cronbach’s α values in this study were 0.82 for verbal victimization, 0.89 for physical victimization, 0.81 for attacks on property, and 0.87 for social manipulation, which showed a high internal consistency.

### 2.5. OVS

The OVS is a 21-item self-report scale that assesses adolescents’ online victimization experiences across general, sexual, and racial domains. It includes four subscales: general online victimization (eight items), sexual online victimization (six items), individual online racial discrimination (four items), and vicarious online racial discrimination (three items). For this study, only the subscales for general online victimization (e.g., “people have posted mean or rude things about me on the internet”) and sexual online victimization (e.g., “people have asked me for sexy pictures of myself online”) of the Chinese version adopted by Law and Fung [5] were used. The respondents rated each item on a 6-point Likert scale, ranging from 1 (never) to 6 (every day), to indicate how often they had experienced online victimization in the previous school term. In this study, the internal consistency was high for both subscales (Cronbach’s α = 0.91 for general online victimization and α = 0.90 for sexual online victimization).

### 2.6. Design

A cross-sectional correlational design was employed in the current study. After collecting the data, hierarchical linear regression analyses were conducted to perform the data analysis. First, age (covariate), sex (with “male” as the baseline), verbal victimization, physical victimization, property attacks, and social manipulation were used to predict general and sexual online victimization (criteria). Then, to examine the moderation effect of sex, interaction terms (sex × four forms of victimization) were included in the model. Mean centering was performed on the four forms of victimization, reducing multicollinearity due to the addition of the moderator and interaction terms. The regression models’ R^2^ and standardized coefficients, β, were recorded.

## 3. Results

The means and standard deviations of the variables for the whole sample and subsamples by sex are presented in Table 1.

### 3.1. Correlation Analysis

Table 2 presents the correlational matrix. The Pearson correlations show that both general online victimization and sexual online victimization were significantly and positively correlated with physical victimization, verbal victimization, social manipulation, and attacks on property (*p* < 0.05).

### 3.2. Hierarchical Linear Regression

#### 3.2.1. General Online Victimization

In Step 1, the model significantly predicted general online victimization, R^2^ = 0.15, F(6, 787) = 22.35, *p* < 0.001. However, the addition of the interaction terms in Step 2 did not significantly increase the variance explained, ΔR^2^ = 0.01, ΔF(4, 783) = 2.27, *p* = 0.06. In the final regression model, sex, verbal victimization, and social manipulation were significant correlates of general online victimization, while age, physical victimization, and attacks on property were not significant. This study showed an absence of multicollinearity because all VIF values were below 5. The final regression model explained 14.6% of the variance (details see Table 3).

#### 3.2.2. Sexual Online Victimization

In Step 1, the model significantly predicted sexual online victimization, R^2^ = 0.09, F(6, 787) = 12.69, *p* < 0.001. Nevertheless, the addition of the interaction terms in Step 2 did not significantly increase the variance explained, ΔR^2^ = 0.01, ΔF(4, 783) = 1.33, *p* = 0.26. In the final regression model, age, sex, physical victimization, and attacks on property were significant correlates of sexual online victimization, while verbal victimization and social manipulation were not significant. All VIF values were less than 5, indicating no signs of multicollinearity. The final regression model accounted for 8.8% of the variance. The details of the final regression models are presented in Table 3.

## 4. Discussion

Both general online victimization and sexual online victimization were significantly and positively correlated with physical victimization, verbal victimization, social manipulation, and attacks on property. The finding indicates that adolescents who experienced various forms of face-to-face victimization in physical settings also experienced victimization online. Victimization appears to affect the same individuals, whether in a physical setting or on the internet. This symbiotic phenomenon could be explained by the self-determination theory and the social compensation hypothesis. Specifically, the experience of face-to-face victimization may diminish victims’ coping skills [29]. The coping strategies an individual employs can influence the level of support they receive during a stressful context [30]. Thus, face-to-face victims may feel a low perception of social support [31] and show a poor social connection in their real lives [31,32]. Additionally, studies show that adolescents who are frequently bullied (e.g., physically, verbally, or financially) may become more vulnerable and have a lower level of competence perception [29]. Thus, they may tend to seek autonomy, competence, and connection in the online world to compensate [18]. However, they may carry offline relationships into the online realm, thereby manifesting face-to-face conflicts or victimization in online spaces where similar social dynamics are replicated [33]. A piece of existing empirical evidence to prove such theoretical explanations is that researchers using a Spanish sample of adolescents found that low social competence, including interpersonal difficulties and social skills, can increase the likelihood of online victimization [34]. The continuity of victimhood has profound negative influences on those adolescents in terms of increasing their severity of depression [35], social withdrawal [36], risk of self-harm [37], suicidal ideation and suicide attempts [38]. Therefore, it is important to help affected adolescents escape the cycle of face-to-face and online victimization.

The present study identified and compared the significant correlates of general online victimization and sexual online victimization, thus increasing our understanding of these high-risk factors. The findings highlight the importance of addressing specific correlates when designing prevention and intervention efforts aimed at reducing general online victimization and sexual online victimization. They indicate that two types of face-to-face victimization—verbal victimization and social manipulation—are significant correlates of general online victimization, but the other two types are not. In contrast, physical victimization and attacks on property were found to be significant correlates of sexual online victimization. What’s more, the findings show that being male is a significant correlate of both general online and sexual online victimization, and older age was a significant correlate with sexual online victimization.

In terms of gender, our findings support the notion that males experience a higher prevalence of both general online victimization and sexual online victimization compared to females, aligning with numerous prior studies [22,39,40]. Our research indicated that male teenagers are more likely to engage in risky and challenging online behaviors, rendering them more susceptible to cyberattacks [41]. Specifically, adolescent males may exhibit more sexual risk behavior online than females [42], placing them at an increased risk of receiving sexual solicitations [43]. Concerning age, our results suggest that within the 12 to 18-year-old age range, individuals of an older age may encounter a higher incidence of sexual online victimization. In other words, sexual online conversations and harassment are more prevalent in late adolescence compared to early adolescence [44,45]. During adolescence, the progression of teenagers’ sexual development and heightened sexual curiosity as they grow older may prompt them to seek sex-related information on the internet more frequently, consequently elevating their risk of online sexual victimization [46]. Moreover, during this specific period, adolescents grapple with increased social and romantic needs and are more susceptible to peer influence. These factors may drive them to access sexual information and engage in related behaviors online, thereby increasing the likelihood of sexual online victimization [47,48].

For general online victimization, the findings suggested that verbal victimization and social manipulation are significant correlates. Adolescents who have experienced verbal victimization and social manipulation in a face-to-face physical environment have a higher risk of being attacked and bullied through text messages, including name-calling [15], spreading rumors, and social exclusion on social media and online platforms [49]. Encountering verbal victimization and social manipulation in real-life situations often indicates that adolescents are facing serious interpersonal conflicts. The findings illustrated that such victims exhibit poor relatedness from the perspective of the self-determination theory, namely the connection and belonging that an individual feels with others [18]. In line with the social compensation hypothesis, adolescents experiencing challenges in social connection are more likely to seek relationship compensation from the online environment, thereby increasing their susceptibility to online victimization [19]. Additionally, face-to-face experiences of relational victimization may have a detrimental impact on adolescents’ mental health, heightening their vulnerability to negative experiences in virtual environments [20]. Some research proposed that psychological trauma might lead to a greater vulnerability to cyberbullying [50]. Thus, parents, teachers, and helping professionals who encounter adolescents experiencing verbal victimization and social manipulation in schools should encourage their conflict resolution, emotion regulation, coping, and communication skills and discourage them from actively participating in posting and sharing personal information or self-disclosure online.

Physical victimization and attacks on property were also found to be significant correlates of sexual online victimization. Compared with verbal victimization and social manipulation, experiences of physical victimization and attacks on property may cause greater psychological harm and emotional disturbance, which can lead to feelings of anxiety, depression, and low self-esteem [51] and, in turn, increase vulnerability to sexual online victimization [23,52,53]. Adolescents who face physical or property harm may have their sense of autonomy and competence undermined, making them feel powerless and vulnerable. Also, the social support they experience in real life (similar to the relatedness highlighted by the self-determination theory) is often insufficient. They may be eager to engage in a romantic relationship online to feel in control and supported. As the social compensation hypothesis showed, adolescent victims of physical victimization and attacks on property in daily life who do not have others’ support or trust in their peers and family members, due to a history of trauma or adverse childhood experiences, may seek help and support from online friends and networks, which can impact their ability to navigate online sexual risks and protect themselves online [54]. Some previous studies have also shown that adolescents who experience physical victimization and attacks on property are particularly vulnerable to sexual online solicitation, which involves an adult or peer seeking sexual contact with a minor online [55], and they are more prone to an elevated risk of sexual online victimization through participating in risky online activities, including disclosing personal information and engaging in sexualized conversations with strangers [56].

Based on the findings and discussions above, several measures can be advocated to protect and prevent youths from both face-to-face and online victimization. Firstly, from an individual perspective, it is imperative to recognize that adolescents undergoing any form of verbal victimization, social manipulation, physical victimization, attacks on property, and general/sexual online victimization face potentially serious consequences for their physical and psychological well-being. It is vital that they receive counselling and psychotherapy to address their trauma, emotional distress, and insecurity in interpersonal relationships both online and offline [57]. Developing their resilience, assertiveness, internal strength, and external support resources is also essential to empower teens in overcoming such victimizations [58]. Secondly, from a cyber environment regulation standpoint, reducing the discussion of sexual topics on online forums, and self-disclosure in the form of photo sharing and sexual conversations, are important for preventing adolescents’ sexual online victimization [59]. Online education and propaganda increasing the awareness of online safety, and resources for healthy sexual behavior and relationships, may also be effective for preventing sexual online victimization [60,61]. Thirdly, from the perspective of community, parents need to provide more parental monitoring and emotional support of adolescents, while schools can focus on establishing mentor–mentee schemes and a harmonious climate, which are crucial for reducing the risk factors [62]. Last but not least, from a social standpoint, revising social policy and legal factors such as laws related to online privacy, cybersecurity, and child protection may also play a role in protecting vulnerable adolescents in the future.

Overall, this study makes meaningful contributions in the practical area of addressing adolescent victimization issues. It found the concurring possibility of face-to-face victimization and online victimization and the moderating factors of age and gender. Based on the findings, prevention, intervention, and policy measures have been put forward from the individual, cyber environment, community, and social perspectives, demonstrating their practicality and significant value.

## 5. Limitations

One of the limitations of the current study was that it was a cross-sectional study in which correlation does not imply causality. Current findings indicated that different forms of face-to-face victimization were distinctively associated with general online victimization and sexual online victimization. However, the mechanisms behind the associations were unclear. Future research could conduct some longitudinal studies or empirical studies with strict experimental designs to further explore the causal relationships and influencing mechanisms between various forms of face-to-face victimizations and online victimizations.

Another limitation was that there were more males than females in our sample. Male teenagers were more willing to participate in our study. Females might be less willing to reveal their sexual online victimization experience. Therefore, whether there were any systematic differences between males and females confounding the results was unknown. Future research could try to exclude the possible impact of gender imbalance through statistical methods or further explore its potential impacts.

Besides, although this study found some significant results, the correlation coefficients of the four types of face-to-face victimization and the two types of online victimization in Table 2 (mostly between 0.2 and 0.35), and the regression coefficients in Table 3 (mostly between 0.08 and 0.19), are small, indicating that the effect size is not large. Future research could improve this problem through various methods, such as more scientific sampling, a larger sample size, a more rigorous survey process, the replacement of appropriate statistical methods, etc.

Finally, our participants were all Hong Kong Chinese. Thus, the current results may not generalize to other cultures. Future studies could investigate if there are any differences in Western cultures.

## 6. Conclusions

This study fills a research gap by comparing the significant correlates of general online victimization and sexual online victimization in relation to four forms of face-to-face victimization. The findings suggested that adolescents with experiences of physical victimization or attacks on property were at higher risk of sexual online victimization, while those with experiences of verbal victimization and social manipulation were significant correlates of general online victimization. Additionally, adolescent boys seem to suffer more from both general and sexual online victimization than girls, and older teens are at greater risk of sexual online victimization. This study generates practical implications for helping professionals and educators involved in developing effective prevention and intervention strategies for adolescents at risk of being victimized.

## Figures and Tables

**Table 1:** Descriptive Statistics for the Variables.

Variable	Male		Female		Total	
	M	SD	M	SD	M	SD
Physical Victimization	1.39	2.42	0.72	1.68	1.13	2.18
Verbal Victimization	1.82	2.48	1.81	2.44	1.81	2.46
Social Exclusion	1.18	2.19	1.15	2.11	1.17	2.16
Attacks on Property	1.16	2.13	0.89	1.60	1.06	1.94
General Online Victimization	12.24	6.47	11.07	4.35	11.78	5.76
Sexual Online Victimization	8.52	4.89	7.63	3.30	8.17	4.35

**Table 2:** Correlation Matrix.

Variable	1	2	3	4	5	6
1. Age	-	-	-	-	-	-
2. Physical Victimization	−0.11 **	-	-	-	-	-
3. Verbal Victimization	−0.06	0.63 **	-	-	-	-
4. Social Exclusion	−0.11 **	0.61 **	0.67 **	-	-	-
5. Attacks on Property	−0.09 *	0.62 **	0.66 **	0.63 **	-	-
6. General Online Victimization	−0.01	0.29 **	0.32 **	0.34 **	0.30 **	-
7. Sexual Online Victimization	0.04	0.24 **	0.20 **	0.22 **	0.25 **	0.65 **

* *p* < 0.05. ** *p* < 0.01.

**Table 3:** Final Regression Models for the Effects of Age, Sex, and Different Types of Peer Victimization on Online Victimization.

Model	Factor	β	*t*	VIF
General Online Victimization	Age	0.03	1.01	1.02
	Sex	−0.09	−2.59 *	1.04
	Physical Victimization	0.05	1.03	2.08
	Verbal Victimization	0.19	3.89 ***	2.18
	Social Exclusion	0.12	2.41 *	2.41
	Attacks on Property	0.06	1.32	2.15
Sexual Online Victimization	Age	0.08	2.27 *	1.02
	Sex	−0.08	−2.17 *	1.04
	Physical Victimization	0.10	2.02 *	2.08
	Verbal Victimization	0.10	1.92	2.18
	Social Exclusion	−0.03	−0.52	2.41
	Attacks on Property	0.15	3.01 **	2.15

* *p* < 0.05. ** *p* < 0.01. *** *p* < 0.001.

## Data Availability

Data are contained within the article.
